# The Successful Treatment of Severe Trigeminal Neuralgia by a Single Mandibular Nerve Block and Subsequent Natalizumab Administration in a Patient with Multiple Sclerosis

**DOI:** 10.7759/cureus.7646

**Published:** 2020-04-12

**Authors:** Ryohei Norioka, Yoko Warabi, Hiroaki Matayoshi, Eiji Isozaki

**Affiliations:** 1 Neurology, Tokyo Metropolitan Neurological Hospital, Tokyo, JPN; 2 Anesthesiology, Tokyo Metropolitan Neurological Hospital, Tokyo, JPN

**Keywords:** multiple sclerosis, trigeminal neuralgia, mandibular nerve block, natalizumab

## Abstract

We report the case of a middle-aged woman who developed trigeminal neuralgia as a sequela of multiple sclerosis (MS). The trigeminal neuralgia was refractory to medications and persisted for two years. Eventually, it was resolved by a mandibular nerve block followed by natalizumab administration. The pain was controlled for 23 months, and additional nerve blocks were not required during this period. It has been previously reported that natalizumab therapy improves the Expanded Disability Status Scale (EDSS) scores and health-related quality of life in patients with MS. In the present case, natalizumab may have prolonged the effect of the mandibular nerve block and consequently improved the patient’s quality of life.

## Introduction

Multiple sclerosis (MS) is a major inflammatory and demyelinating disease of the central nervous system and is associated with autoimmune mechanisms. Trigeminal neuralgia can often complicate the clinical course of MS [1]. The pathophysiological cause of trigeminal neuralgia remains unknown [2]. However, it is believed to be caused by demyelination of the trigeminal nerve projections within the brainstem or chronic inflammatory processes involving the central nervous system [3,4]. Trigeminal neuralgia results in intense pain and affects the Expanded Disability Status Scale (EDSS) scores and health-related quality of life. When it occurs in patients with MS, it is generally refractory to drugs and requires multiple surgical procedures [5].

Here, we describe the successful treatment of debilitating drug-refractory trigeminal neuralgia by a single mandibular nerve block followed by natalizumab administration in a middle-aged woman with MS.

## Case presentation

**Figure 1:**
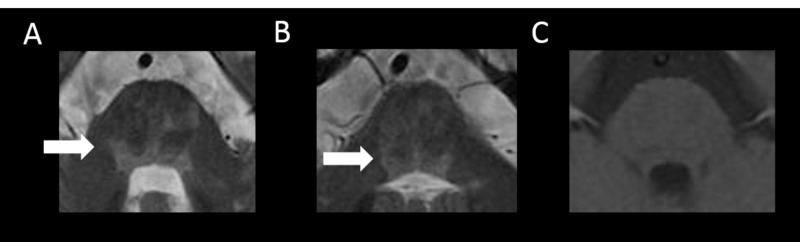
Brain MRI findings - lesions in the brainstem A, B: brain T2-weighted imaging shows multiple high-intensity lesions in the brainstem, including the intramedullary running path of the trigeminal nerve (white arrows); C: the lesions exhibit no gadolinium enhancement MRI: magnetic resonance imaging

A 48-year-old woman was admitted to our hospital due to drug-refractory trigeminal neuralgia. At 31 years of age, she had experienced double vision and tetraparesis after giving birth to her second child. Since then, she had experienced relapses twice a year and had been diagnosed with relapsing-remitting MS. She had been prescribed interferon b-1b; however, she could not continue the treatment because of the onset of depression. Since interferon b-1b had been the only disease-modifying drug sold at that time, her treatment had been switched to prednisolone (15 mg/day) and azathioprine (50 mg/day). This treatment had been ineffective, and she had continued to experience relapses twice a year. At 43 years of age, she was confined to a wheelchair. At 46 years of age, she had developed drug-refractory trigeminal neuralgia with demyelinating brainstem lesions that caused sustained, strong right-sided pain at the back of the tongue and consequently affected her oral intake and daily life activities. An MRI showing the extent of her brainstem lesions is shown in Figure 1A and Figure 1B. Because she experienced paroxysmal pain more than a dozen times a day in addition to persistent pain, she had decreased her food intake and lost weight.

At 48 years of age, the patient was admitted to our hospital. Neurological examination revealed drug-refractory trigeminal neuralgia with strong right-sided pain at the back of the tongue, lower lip, and chin; right-sided hypoesthesia in the lower lip; diplopia; bilateral medial longitudinal fasciculus (MLF) syndrome; moderate spastic tetraparesis; moderate ataxia; and paresthesia and hypoesthesia in the right upper and both lower extremities. Her blood tests were negative for inflammatory markers and autoantibodies, including anti-aquaporin 4, anti-nuclear, and anti-dsDNA antibodies. The cerebrospinal fluid analysis revealed a normal white blood cell count (0/µL), protein level (29 mg/dL), and myelin basic protein level (<31.2 pg/mL), although the immunoglobulin G index was elevated (0.87) and oligoclonal IgG bands were present.

Brain T2-weighted imaging demonstrated multiple high-intensity lesions in the brainstem, including the intramedullary running path of the trigeminal nerve. The lesions exhibited no gadolinium enhancement (Figure 1A-1C).

**Figure 2:**
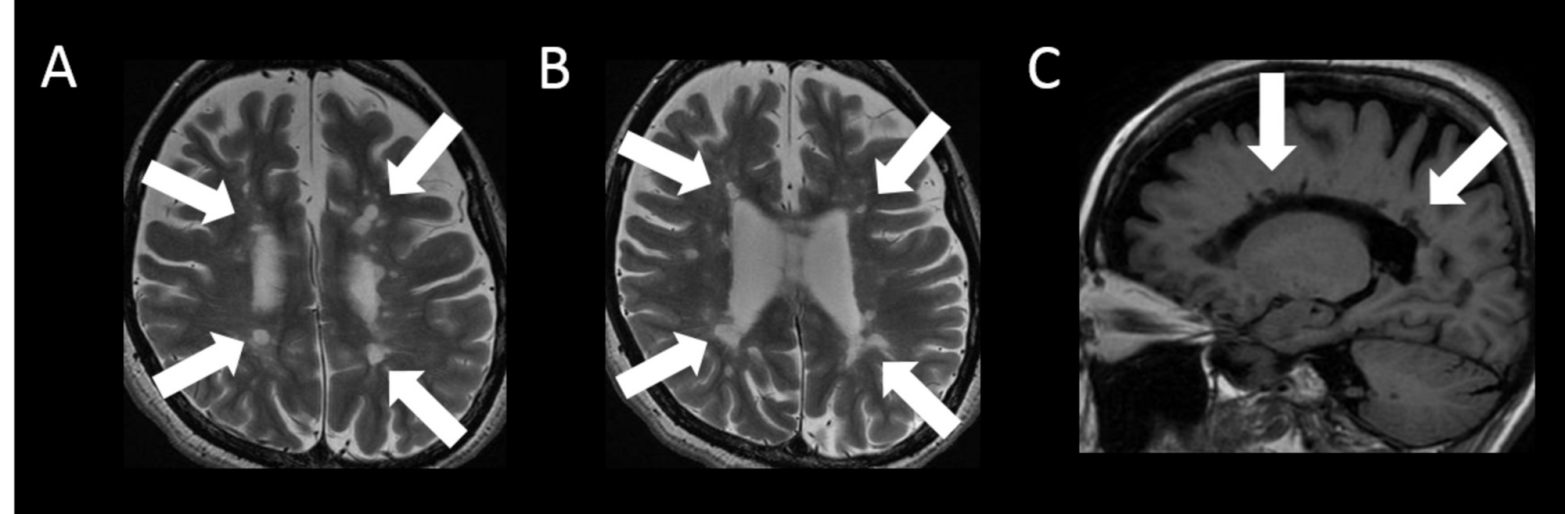
Brain MRI findings - lesions in the periventricular and subcortical white matter A, B: brain T2-weighted imaging shows high-intensity lesions in the periventricular and subcortical white matter (white arrows); C: T1-weighted imaging shows that many of these lesions are hypointense (white arrows) MRI: magnetic resonance imaging

The lesions involved the right trigeminal nerve tract, but vascular compression at the entry of the trigeminal nerve root was not recognized on fast imaging employing steady-state acquisition (FIESTA). In addition, brain T2-weighted imaging showed high-intensity lesions in the periventricular and subcortical white matter. T1-weighted imaging showed that many of these lesions were hypointense (Figure 2A-2C).

**Figure 3:**
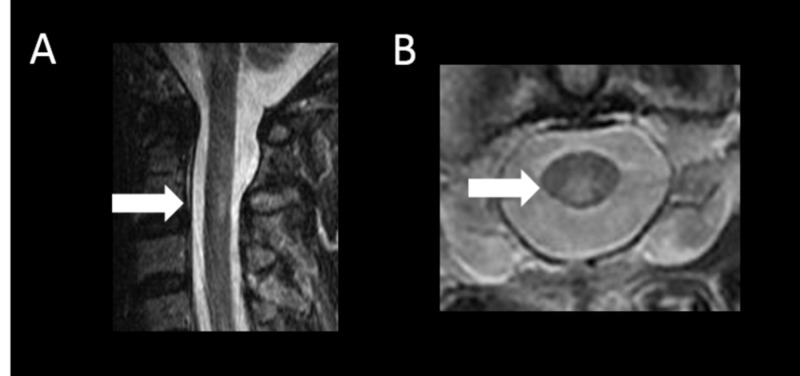
Spinal cord MRI findings A, B: spinal cord T2-weighted imaging shows 11 high-intensity white matter lesions with a length equivalent to less than 1.5 vertebral bodies (white arrows) MRI: magnetic resonance imaging

Spinal cord T2-weighted imaging showed 11 high-intensity white matter lesions with a length equivalent to less than 1.5 vertebral bodies (Figure 3A, 3B).

The patient was diagnosed with trigeminal neuralgia due to relapse of MS. The trigeminal neuralgia was refractory to medications such as carbamazepine, levetiracetam, acetaminophen, tramadol, and morphine. Therefore, an anesthesiologist administered a mandibular nerve block with radiofrequency thermocoagulation. The needle was inserted at an extraoral point 4 cm from the corner of the mouth and advanced to the foramen ovale. Coagulation was performed at 80 °C for 180 s after a trial with a local anesthetic. Her pain resolved (the Numeric Pain Rating Scale score improved from 8-10 to 0-1) [6]. She resumed oral intake despite the presence of a nasogastric tube inserted after admission. Three months later, we initiated natalizumab treatment after confirming that she was negative for the anti-John Cunningham virus.

**Figure 4:**
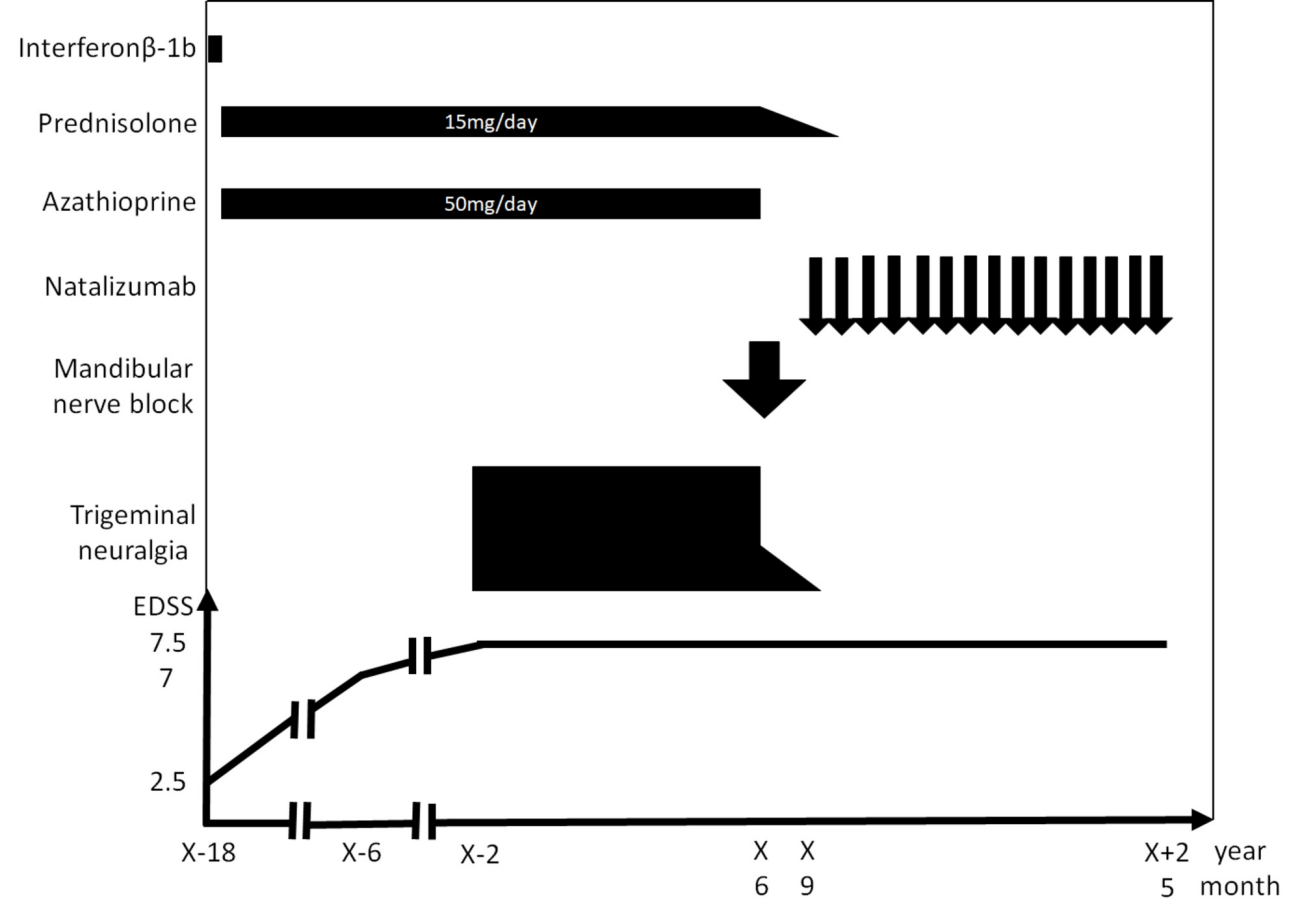
Summary of the clinical course After medications proved ineffective for trigeminal neuralgia, a mandibular nerve block was administered and was found to be effective. Three months after the block, natalizumab treatment was initiated, which controlled the pain without the requirement of additional blocks for 23 months EDSS: Expanded Disability Status Scale

Natalizumab treatment was continued without additional nerve blocks, and she did not experience pain or develop new demyelinating lesions for 23 months (Figure 4).

## Discussion

We derived two important findings from the present case. First, a single mandibular nerve block could resolve the pain of trigeminal neuralgia associated with MS with no relapses for 23 months. Second, natalizumab treatment may have prolonged the pain-relieving effects of the nerve block, thus resulting in an improved quality of life.

Although a single nerve block can result in an improvement in approximately 95% of the patients, it should be noted that the effect of mandibular nerve block is not permanent [7]. Moreover, unlike classical trigeminal neuralgia, MS-related trigeminal neuralgia tends to respond poorly to medications and requires multiple surgical procedures including the nerve block, with repeat surgery often necessary within one year [5]. Accordingly, the present case is clinically significant, considering that the patient remained pain-free for 23 months after a single mandibular nerve block.

The pathophysiology of trigeminal neuralgia remains unclear, although the main theory is that chronic inflammatory processes in the central nervous system cause secondary trigeminal neuralgia, including that associated with MS [4]. In this case, three months before natalizumab treatment, the patient had received radiofrequency thermocoagulation, which is very effective for symptomatic control of trigeminal neuralgia. Additionally, natalizumab treatment may have prolonged the pain-relieving effects of the nerve block for 23 months. Natalizumab inhibits the migration of inflammatory cells across the blood-brain barrier by preventing the adhesion of integrin to its ligands on the vascular endothelium in the brain. It also inhibits cerebral leukocyte invasion and neurotoxic cytokine production, thus contributing to the suppression of inflammation and white matter lesions [8,9]. Inhibition of inflammation by natalizumab can lead to resolution of focal edema and, consequently, neuralgia. However, azathioprine and disease-modifying drugs other than natalizumab will not inhibit the migration of inflammatory cells across the blood-brain barrier. Therefore, these drugs might not prolong the pain-relieving effect.

Although disease-modifying drugs such as natalizumab are widely used and work by interacting with the immune system, they are not routinely used for symptomatic treatment of sequelae that often occur as a result of MS. To date, no study has evaluated the effects of immunotherapy for trigeminal neuralgia. However, natalizumab treatment reportedly decreases the relapse rate and stabilizes disability levels in MS [10]. In addition, it improves EDSS scores and health-related quality of life [11,12]. In Japan, natalizumab is used to prevent relapses and suppress the progression of physical disability. Although we considered that our patient had transitioned to a secondary progressive phase, her trigeminal neuralgia was associated with a relapse of inflammatory demyelination. We initiated natalizumab treatment after considering the fact that no disease-modifying drugs were used.

## Conclusions

The findings from this case suggest that mandibular nerve block followed by natalizumab therapy is effective in controlling MS-associated trigeminal neuralgia, which, to date, has been generally treated with combination treatment involving carbamazepine, nerve blocks, and surgery.

## References

[1] Montano N, Papacci F, Cioni B, Di Bonaventura R, Meglio M (2013). What is the best treatment of drug-resistant trigeminal neuralgia in patients affected by multiple sclerosis? A literature analysis of surgical procedures. Clin Neurol Neurosurg.

[2] Love S, Coakham HB (2001). Trigeminal neuralgia: pathology and pathogenesis. Brain.

[3] Jensen TS, Rasmussen P, Reske-Nielsen E (1982). Association of trigeminal neuralgia with multiple sclerosis: clinical and pathological features. Acta Neurol Scand.

[4] Zakrzewska JM (2002). Diagnosis and differential diagnosis of trigeminal neuralgia. Clin J Pain.

[5] Krishnan S, Bigder M, Kaufmann AM (2018). Long-term follow-up of multimodality treatment for multiple sclerosis-related trigeminal neuralgia. Acta Neurochir (Wien).

[6] Jensen MP, McFarland CA (1993). Increasing the reliability and validity of pain intensity measurement in chronic pain patients. Pain.

[7] Yadav YR, Nishtha Y, Sonjjay P, Vijay P, Shailendra R, Yatin K (2017). Trigeminal neuralgia. Asian J Neurosurg.

[8] Liesz A, Zhou W, Mracskó É (2011). Inhibition of lymphocyte trafficking shields the brain against deleterious neuroinflammation after stroke. Brain.

[9] Brown BA (2009). Natalizumab in the treatment of multiple sclerosis. Ther Clin Risk Manag.

[10] Butzkueven H, Kappos L, Pellegrini F (2014). Efficacy and safety of natalizumab in multiple sclerosis: interim observational programme results. J Neurol Neurosurg Psychiatry.

[11] Phillips JT, Giovannoni G, Lublin FD (2011). Sustained improvement in Expanded Disability Status Scale as a new efficacy measure of neurological change in multiple sclerosis: treatment effects with natalizumab in patients with relapsing multiple sclerosis. Mult Scler.

[12] Rudick RA, Miller D, Hass S (2007). Health-related quality of life in multiple sclerosis: effects of natalizumab. Ann Neurol.

